# Molecular Dynamics Simulations of Ion Drift in Nanochannel Water Flow

**DOI:** 10.3390/nano10122373

**Published:** 2020-11-28

**Authors:** Filippos Sofos, Theodoros Karakasidis, Ioannis E. Sarris

**Affiliations:** 1 Physics Department, University of Thessaly, 35100 Lamia, Greece; fsofos@uth.gr; 2 Department of Mechanical Engineering, University of West Attica, 12244 Athens, Greece; sarris@uniwa.gr

**Keywords:** nanochannel flows, electric field, ion separation, molecular dynamics

## Abstract

The present paper employs Molecular Dynamics (MD) simulations to reveal nanoscale ion separation from water/ion flows under an external electric field in Poiseuille-like nanochannels. Ions are drifted to the sidewalls due to the effect of wall-normal applied electric fields while flowing inside the channel. Fresh water is obtained from the channel centerline, while ions are rejected near the walls, similar to the Capacitive DeIonization (CDI) principles. Parameters affecting the separation process, i.e., simulation duration, percentage of the removal, volumetric flow rate, and the length of the nanochannel incorporated, are affected by the electric field magnitude, ion correlations, and channel height. For the range of channels investigated here, an ion removal percentage near 100% is achieved in most cases in less than 20 ns for an electric field magnitude of E = 2.0 V/Å. In the nutshell, the ion drift is found satisfactory in the proposed nanoscale method, and it is exploited in a practical, small-scale system. Theoretical investigation from this work can be projected for systems at larger scales to perform fundamental yet elusive studies on water/ion separation issues at the nanoscale and, one step further, for designing real devices as well. The advantages over existing methods refer to the ease of implementation, low cost, and energy consumption, without the need to confront membrane fouling problems and complex electrode material fabrication employed in CDI.

## 1. Introduction

An investigation of fluid flows at the micro- and/or the nanoscale has attached technological and scientific importance during the past decades due to the increasing number of devices operating at small scales. Flows in such conditions are faced with the high surface area-to-volume ratio, the breakdown of the continuum hypothesis of the no-slip condition, and the strong effect of fluid/wall interactions. The effect of walls in a miniaturized system is strong and expands over a region of 1–2 nm close to the wall, affecting the resulting flow and transport properties, while in larger dimensions, these properties approach the bulk behavior [1,2,3,4].

Among novel applications of fluid flows along nanochannels, water desalination and purification methods play a significant role for the fabrication of nanofiltering devices, which are capable of removing or blocking unwanted substances from the solution. As water resources have been decreasing worldwide, fresh water supply from seawater seems as the only choice to provide drinking water for people, especially in developing countries [5,6]. Understanding the mechanism of water and ion transport inside nanochannels is the key to suggest an effective method for water purification and desalination that could achieve a high flux of clean water in a practical and cost-efficient manner [7,8]. Different methods, novel materials, and flow-controlling driving forces have been suggested.

Within the sub-nanometer range, theoretical and experimental studies of water flows forming filtration membranes have shown promising capabilities [9,10,11]. The choice of materials incorporated is highly important. It has been found both with theoretical and experimental evidences that nanochannel membranes made of carbon nanotubes (CNTs), graphene or zeolites, have shown remarkable performance and achieved high flux rates due to the nature of the wall/fluid interaction [12,13,14,15]. Desalination membranes are also inspired from biological systems, such as aquaporins, where water permeation is affected by the application of DC or AC electric fields of various frequencies [16,17,18,19]. Recently, efforts have been devoted to the fabrication of nanomaterials such as black phosphorus (BP), which has been introduced as one of the next promising candidates to replace graphene in medical and environmental applications [20,21].

According to the separation process, membrane methods include microfiltration (MF), ultrafiltration (UF), nanofiltration (NF), reverse osmosis (RO), forward osmosis (FO), pervaporation, and membrane distillation (see [22] for more details). Currently, RO technologies are broadly used in practical, large-scale applications of removing unwanted substances from sea or brackish water [23]. Notwithstanding its commercialization, RO faces the challenge of membrane fouling, high energy consumption, and brine production management [24]. Advancing toward more energy-efficient water desalination systems, trading off between kinetics and energetics, the Capacitive DeIonization (CDI) technology has been introduced as an emerging ion removal technique that has not yet achieved widespread application in large-scale systems [25,26].

CDI is broadly defined as a cost-effective process, especially at small scales, which is based on the electrosorption of cations and anions on charged electrode surfaces leading to ion rejection [27]. An electric field, usually between 0.01 and 2.0 V [28,29,30], is exploited between two electrodes forming a channel with upper and lower boundaries, where a sequential charge/discharge process can lead ions through the electrodes and reject them. Different types of electrode materials have been incorporated for CDI. For example, graphene surfaces have shown good salt removal ability due to their unique physicochemical properties [31]. High porosity and a cost-effective fabrication of activated carbon clothes have also been considered in recent CDI applications [32].

Recent computational studies, comparable to CDI, report increased ion removal either at the nanoscale [33] or at the macroscale [34]. In the present paper, by combining fundamental insights both from water flows at the nanoscale and CDI techniques, a new ion separation method is proposed with Molecular Dynamics (MD) simulations [35,36,37,38]. The main idea behind CDI, i.e., ion movement toward the walls, has been achieved with the application of an external electric field, equally applied on all system particles. The electric field, apart from controlling ion separation, seems to alter water molecule properties as it affects their orientation. Furthermore, water flows inside nano-conduits, a common practice in membrane separation techniques where flow is driven by a parallel to the flow electric field, is also incorporated [39].

Nevertheless, water molecules, along with Na^+^ and Cl^−^ ions, flow inside a nanochannel driven by an external body force, analogous to a pressure gradient, in perpendicular to the electric field direction. Ions are drifted along the channel until the effect of the electric field becomes strong enough to lead them near the walls. Simulation ends when clean water flows around the channel centerline and ions have approached the carbon walls, and this takes less than *t* = 20 ns in this system. Dimensions of the channels investigated cover the range *h* = 1.5–13.5 nm, and electric field values are between *E* = 0.1–3.0 V/Å, while ion concentration (mass percentage) for a NaCl solution lies in the range *c* = 1.35–1.39%. The choice of the simulated system parameters is made so that it becomes possible to report on both nanoscale desalination processes and larger systems at the micro/mini/macro scale, having in mind that nanoscale wall effects diminish as channel dimensions increase [1,40].

The proposed configuration presents no fouling issues, demands no special wall/electrode material, and achieves satisfactory ion removal in solutions with salt concentration similar to seawater.

## 2. Materials and Methods

### 2.1. Channel Description

**Figure 1:**
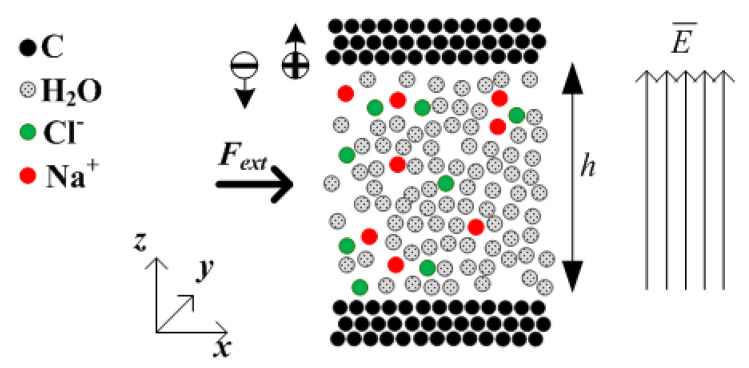
Simulated channel model with water and ions flowing among carbon walls. The direction of the electric field applied is perpendicular to the external driving force.

A periodic box of water molecules flowing between two planar, solid, infinite, atomistic, carbon walls, forming a Poiseuille-like nanochannel (Figure 1), is considered for the simulations. To investigate salt ion removal, Na^+^ and Cl^−^ ions are also added in the flow region in a random manner. The box is periodic at the *x-*, *y-* and fixed at the *z-*direction. Channels of various heights (i.e., the distance between the walls) are simulated spanning the range *h* = 1.5–13.5 nm. Channel dimensions and the respective number of particles are given in Table 1.

Wall particles are initially arranged on a face-centered cubic (fcc-101) lattice, which is a common initial configuration for walls in MD simulations (see, for example, [41]), and tether around their equilibrium position due to an applied spring force $F=-K\left(r(t)-r_{eq}\right)$, where *r*(*t*) is the vector position of a wall particle at time *t*, *r_eq_* is its initial lattice position vector, and *K* = 50.0 Kcal/molÅ^2^ is the spring constant. An external driving force $F_{ext}$ is applied along the *x*-dimension to every fluid particle during the simulations. Wall atoms absorb the increase in fluid kinetic energy and Nosé–Hoover thermostats [42,43] at the thermal walls are employed in order to keep the system’s temperature constant, at *T* = 300 K.

Lennard–Jones (LJ) parameters are assigned to wall and fluid particles. The LJ potential between two particles *i* and *j* is described by the equation

(1) $$u(r_{ij})=\{\begin{array}{cc} 4\varepsilon \left[{\left(\frac{\sigma }{r_{ij}}\right)}^{12}-{\left(\frac{\sigma }{r_{ij}}\right)}^{6}\right] & ,r_{ij}<r_{c}\\ 0 & r_{ij}\geq r_{c} \end{array}$$

where parameter *ε* indicates the interaction strength, *σ* defines the length scale, and *r_c_* represents the cut-off radius. Moreover, the Coulombic interaction for charged atoms is given by

(2) $$V_{C}=\frac{Cq_{i}q_{j}}{e_{r}},r<r_{c}$$

where *C* is the energy conversion constant, *q_i_* and *q_j_* are the charges, and *e* is the dielectric constant. The cut-off radius is *r_c_* = 9 Å for both LJ and Coulombic interactions. Potential parameters and masses for all atomic pairs are summarized in Table 2. The parameters *ε* and *σ* for two different species *α* and *b* are obtained by the Lorentz–Berthelot mixing rule $\sigma_{ab}=\left(\sigma_{a}+\sigma_{b}\right)/2$ and $\varepsilon_{ab}=\sqrt{\left(\varepsilon_{a}\varepsilon_{b}\right)}$, based on the self-interaction parameters given in this table. Thus, Lorentz–Berthelot combining rules are applied to the ion–ion and water–water interactions. This force field is quite popular in studies of NaCl in water [44,45].

The SPC/E (extended simple point charge) pair potential is chosen for our water model for its simplicity, computational speed, and efficiency compared to experimental results [33,46,47,48]. Water is expressed as a 3-site rigid molecule with charge *q*_H_ = 0.4238 for hydrogen and *q*_O_ = −0.8476 for oxygen. Water bonds are constrained using the SHAKE algorithm as implemented in Large-scale Atomic/Molecular Massively Parallel Simulator (LAMMPS) [49], where the equilibrium O–H bond length and the H–O–H angle are 1 Å and 109.47°, respectively. Bond and angle styles are harmonic; after each SHAKE algorithm application, they return to their equilibrium values. Flow is induced and driven by an external force *F_ext_* = 0.025 kcal/molÅ applied to each one of the *N* fluid particles on the *x*-direction.

At the beginning of the simulations, initialization runs for about 5 ns time take place. From this point onwards, the external driving force and the electric field are applied to the flow, and results are saved and averaged for three or four consecutive runs of 5 ns each (larger channels demand more time to reach steady flow). This simulation duration is expected to yield equilibrated results, as also shown in another studies [50,51,52].

### 2.2. Ion Separation

**Figure 2:**
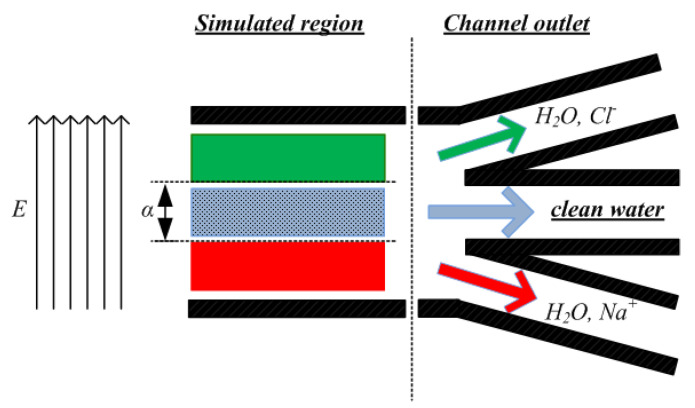
Proposed desalination channel geometry. Clean water flows around the channel centerline (*a* region), while ions have moved near the walls.

Figure 2 presents a desalination configuration that could be used in order to remove ions from water. The internal region is where clean water flows and its width is characterized by the variable *a*, where *a* = 40%*h* (values are given in Table 1). In this work, simulations are carried out for the Poiseuille-like part of the channel; the outlet region will be included in a future work, and it is shown here to support our method description. A similar configuration has also been used at the macroscale [34]. To achieve ion separation from water, an external electric field *E* is applied (it could be seen as a field originating from the homogeneous distribution of opposite sign charges on the two opposite nanochannel wall surfaces), resulting in an electric force *F_e_* = *qE* acting on the *z*-dimension, perpendicular to the flow [50] (direction shown in Figure 1). The model shown presents similarities with the CDI method; however, there are some basic differences.

Walls in the present case are impermeable, i.e., ions cannot flow through them, and they are not transformed to electrodes. As implemented in LAMMPS, the electric field is assigned to the whole simulation box (on the *z*-direction, see direction in Figure 1). Na^+^ and Cl^−^ ions move toward each sidewall, due to the application of the electric force *F_e_*, and simultaneously streamwise similar to water molecules under the effect of the external driving force, *F_ext_*. As long as ions approach the walls, their removal is facilitated at the channel outlet.

Values of *E* investigated here are in the range *E* = 0.1–3.0 V/Å, such that the total simulation time is reduced to 5–20 ns, as already pointed out. The aim of this work is to investigate the electric field effect on a wider range of values. Attention has also been drawn to the fact that in the frame of classical MD simulations, no chemical reactions take place; the electric field corresponds to the external field applied as the gradient of voltage applied on the walls across the nanochannel *z*-direction.

## 3. Results

Na^+^ and Cl^−^ ions are randomly located between water molecules. Since the external electric field acts on all particles, negatively charged particles are expected to migrate toward the lower wall, and positively charged particles are expected to migrate toward the upper wall. Figure 2 reveals the desired outcome of the simulations. Pure water is expected to flow in the middle channel region of width *a* = 40%*h*, while Na^+^ and Cl^−^ ions are localized near the walls, inside the two “reject” outlet regions. The ratio of ions localized in the two “reject” regions is considered as the percentage of ion removal.

Next, investigations concerning different channel widths and various values of the applied electric field are carried out, so that the percentage of ions to be removed is estimated.

### 3.1. Effect of the Channel Width

Density profiles in nanochannels reveal density variations that develop in the fluid due to the presence of the walls. The instantaneous particle number density N^*^ is calculated by counting the number of particles *N_bin_* located in each simulation bin at the current timestep, divided by the total particle number *N* inside the channel, as $N^{*}=N_{bin}/N$. This number, different for each particle, is averaged over successive parts of the total simulation duration to achieve statistical accuracy.

**Figure 3:**
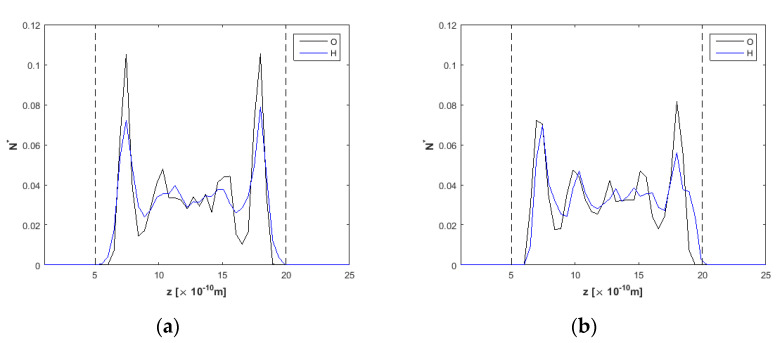
Density profiles for O and H, *F_ext_* = 0.025 kcal/molÅ and *h* = 1.5 nm, (**a**) at *t* = 5 ns, *E* = 0.0 V/Å and (**b**) *t* = 20 ns, *E* = 2.0 V/Å. Dashed lines denote wall limits.

**Figure 4:**
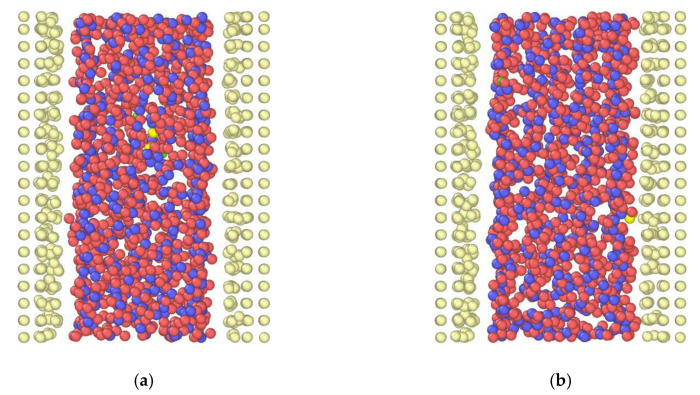
Simulation snapshots for the *h* = 1.5 nm channel, (**a**) at *t* = 5 ns, *E* = 0.0 V/Å and (**b**) *t* = 20 ns, *E* = 2.0 V/Å. Particles are: H—pink, O—blue, C—white, Na^+^—yellow, Cl^−^—green. *z* = 0—left side, *z* = 25—right side, as in Figure 3.

Starting from the *h* = 1.5 nm channel, oxygen and hydrogen density profiles are presented in a simulation run with no external electric field applied and an external force *F_ext_* = 0.025 kcal/molÅ at the *x*-direction to drive the flow in Figure 3a. Density profiles present high peaks for oxygen near the walls, while hydrogen peaks are lower. According to [53], water density profiles present inhomogeneity due to the presence of ions and the electric field (which acts as there are two charged electrodes in the walls’ positions). After the application of an electric field *E* = 2.0 V/Å (Figure 3b), it is observed that oxygen peaks near the upper and lower wall decrease in height compared to the *E* = 0 V/Å case. Hydrogen atoms (positive charge) approach closer to the upper wall than oxygen atoms. According to the electric field direction (see Figure 1), the topmost wall layer (*z* = 20) acts as negatively charged, while the bottommost wall (*z* = 0) acts as positively charged. Near the upper wall, it appears that the orientation of water molecules changes significantly, as hydrogen atoms point toward the carbon wall. On the opposite wall, hydrogen atoms are detracted from the positive charge of the wall, and the respective density peak is located further from the wall compared to the oxygen peak. All the above can also be inferred by the simulation snapshots from OVITO [54] in Figure 4. More specifically, Figure 4b reveals the orientation of water molecules, as H are attracted to the right wall and detracted from the left wall. Similar observations were found in [50].

**Figure 5:**
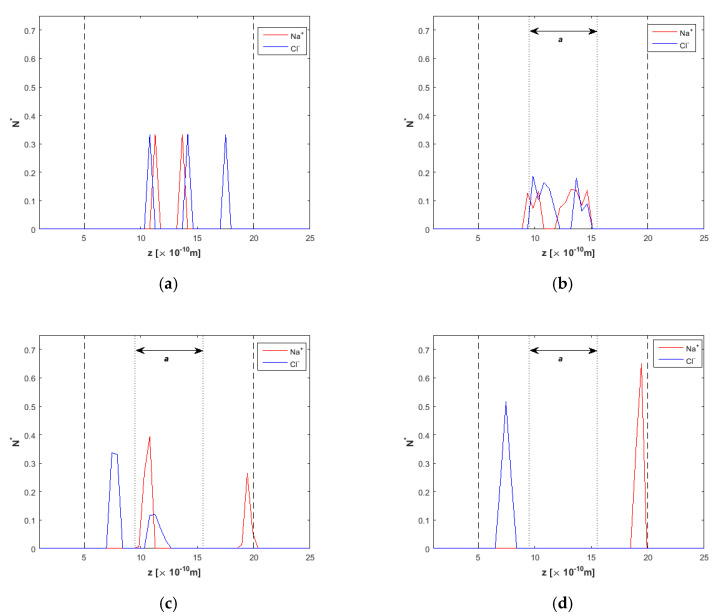
Ion concentration across the *h* = 1.5 nm nanochannel at various times of the simulation, *F_ext_* = 0.025 kcal/molÅ, (**a**) *t* = 5 ns, *E* = 0.0 V/Å, (**b**) *t* = 8 ns, *E* = 2.0 V/Å, (**c**) *t* = 15 ns, *E* = 2.0 V/Å, and (**d**) *t* = 20 ns, *E* = 2.0 V/Å. Dashed lines denote wall limits and dotted lines enclose the desired clean water region.

Ion concentration at various simulation times is presented in Figure 5a–d. Before the electric field is applied, in Figure 5a, non-symmetric ion profiles are obtained due to the small ion concentration in the mixture and their initial random establishment, which limits somehow the statistical accuracy. However, ions are localized around the centerline, away from the walls. After 8 ns (Figure 5b), ions have not moved toward the wall and are still located inside the “clean water” region between the two dotted lines (see Figure 2 for details). At *t* = 15 ns, ion separation has started (Figure 5c), and at *t* = 20 ns (Figure 5d), it seems that ions have approached the two “ion-reject” regions near the walls, leaving clean water to flow in the *a* region.

Another point worth mentioning is the difference in Na^+^ and Cl^−^ peaks in Figure 5d, as Na^+^ approaches closer to the top wall compared to the respective Cl^−^ peak on the opposite bottom wall. This behavior is attributed to wall wettability properties, which is pronounced in channels of this height range [55]. Taking in mind ion and wall *ε* properties (Table 2), we define the ratio $\varepsilon_{r}=\varepsilon_{Na-C}/\varepsilon_{Na-Na}$ to characterize the wall as Na-ionophobic ($\varepsilon_{r}<1$) or Na-ionophilic ($\varepsilon_{r}\geq 1$), according to [56]. In this work, we have $\varepsilon_{r-Na}=0.3035$ (the wall is Na-ionophobic) and $\varepsilon_{r-Cl}=1.1761$ (the wall is Cl-ionophilic). It has been found that hydrophobic walls lead to sharper density peaks, while smoother peaks are usually observed near hydrophilic walls [55].

**Figure 6:**
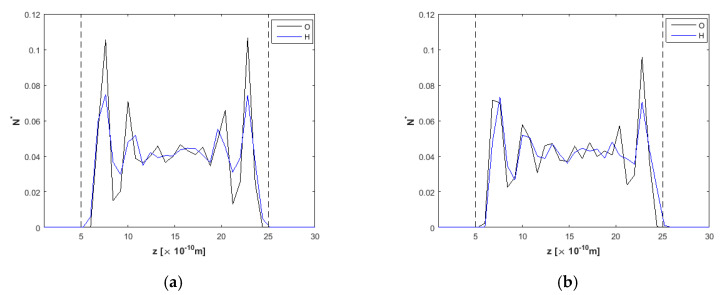
Density profiles for O and H, *F_ext_* = 0.025 kcal/molÅ and *h* = 2.0 nm, (**a**) at *t* = 5 ns, *E* = 0.0 V/Å and (**b**) at *t* = 20 ns, *E* = 2.0 V/Å. Dashed lines denote wall limits.

As for the *h* = 2.0 nm nanochannel, oxygen and hydrogen density profiles are presented in Figure 6. For *E* = 0.0 V/Å, density profiles have the expected shape, with symmetrical peaks for oxygen and hydrogen near the walls, as has been also shown for the *h* = 1.5 nm channel in Figure 3a. In Figure 3b, after *t* = 20 ns, at *E* = 2.0 V/Å, water molecules orientation is also taking place, with the H-peak on the right (top) wall approaching the wall and the respective H-peak on the left (bottom) wall moving slightly away from the wall.

**Figure 7:**
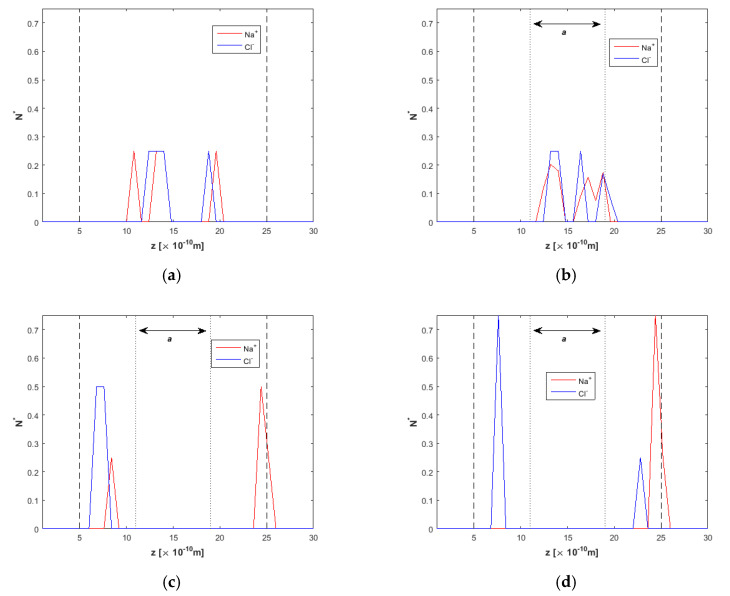
Ion concentration across the *h* = 2.0 nm nanochannel at various times of the simulation, *F_ext_* = 0.025 kcal/molÅ, (**a**) *t* = 5 ns, *E* = 0.0 V/Å, (**b**) *t* = 10 ns, *E* = 2.0 V/Å, (**c**) *t* = 15 ns, *E* = 2.0 V/Å, and (**d**) *t* = 20 ns, *E* = 2.0 V/Å. Dashed lines denote wall limits and dotted lines enclose the desired clean water region.

The density/concentration profiles for Na^+^ and Cl^−^ ions in various simulation timesteps are shown in Figure 7. Ions take place around the channel centerline, while *E* = 0 (Figure 7a). At *t* = 10 ns (Figure 7b), the effect of the electric field is weak, as ions have not moved toward the two reject regions. At *t* = 15 ns, ion removal from the clean water region has reached 100% (Figure 7c). Nevertheless, Na^+^ and Cl^−^ ions have not been completely separated from each other. There are both Na^+^ and Cl^−^ ions near the bottom wall, and even at *t* = 20 ns (Figure 7d), Na^+^ and Cl^−^ ions are correlated near the upper wall. This phenomenon can be explained due to the opposite charge between them, which seems to override the effect of the external electric field in some cases, at least for *E* = 2.0 V/Å.

However, attention has to be drawn to the fact that the desired ion removal from the clean water region is accomplished in any case.

**Figure 8:**
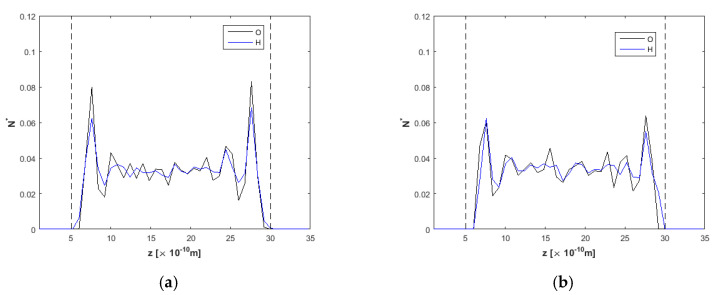
Density profiles for O and H, *F_ext_* = 0.025 kcal/molÅ and *h* = 2.5 nm, (**a**) at *t* = 5 ns, *E* = 0.0 V/Å and (**b**) at *t* = 20 ns, *E* = 2.0 V/Å. Dashed lines denote wall limits.

Density profiles for O and H across the *h* = 2.5 nm nanochannel (Figure 7) present similarities to those shown before for the *h* = 2.0 nm nanochannel (Figure 6). Equally, Figure 8a shows profiles with increased ordering near the walls and lower peaks in the channel interior for *E* = 0.0 V/Å. Oxygen and hydrogen profiles when *E* = 2.0 V/Å are almost identically shaped for this channel width, although the hydrogen profile is slightly shifted to the right due to the water molecules orientation, as explained in the previous paragraphs.

**Figure 9:**
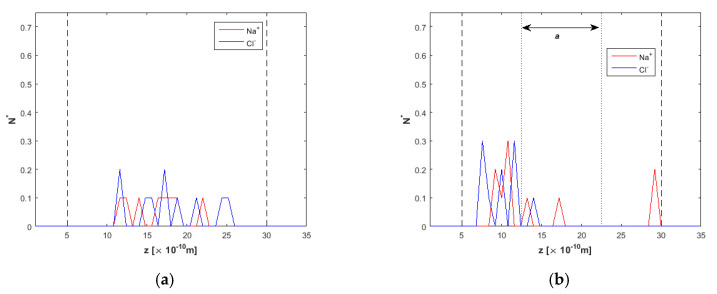

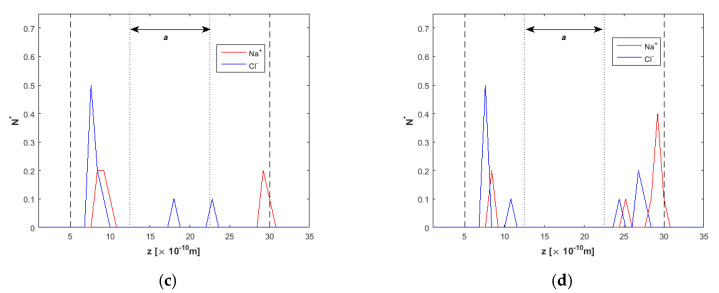
Ion concentration across the *h* = 2.5 nm nanochannel at various times of the simulation, *F_ext_* = 0.025 kcal/molÅ, (**a**) *t* = 5 ns, *E* = 0.0 V/Å, (**b**) *t* = 10 ns, *E* = 2.0 V/Å, (**c**) *t* = 15 ns, *E* = 2.0 V/Å, and (**d**) *t* = 20 ns, *E* = 2.0 V/Å. Dashed lines denote wall limits and dotted lines enclose the desired clean water region.

The estimated ion removal from the channel interior can be concluded from the respective density profiles in Figure 9. Total ion removal is observed after *t* = 20 ns (Figure 9d), although without clear distinction between Na^+^ and Cl^−^ ions near the walls. It is also evident that stronger correlations between Na^+^ and Cl^−^ ions do exist for this channel width.

### 3.2. Effect of the Electric Field Magnitude

To scrutinize the effect of the magnitude of the external electric field applied throughout the simulations, an *h* = 13.5 nm nanochannel is employed for a simulation duration of *t* = 15 ns and an external driving force at the *x*-direction *F_ext_* = 0.025 kcal/molÅ. The wall effect is weaker on the fluid properties in this channel dimension compared to channels studied in the previous section, and electric properties are expected to mainly characterize the ion separation process.

**Figure 10:**
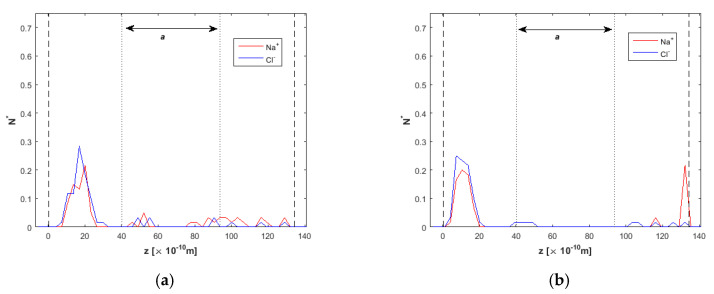

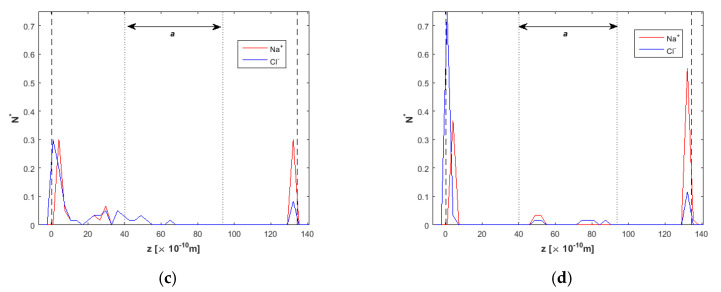
Ion concentration across the *h* = 13.5 nm nanochannel for various values of the external electric field at simulation time *t* = 15 ns, *F_ext_* = 0.025 kcal/molÅ, (**a**) *E* = 0.1 V/Å, (**b**) *E* = 1.0 V/Å, (**c**) *E* = 2.0 V/Å and (**d**) *E* = 3.0 V/Å. Dashed lines denote wall limits and dotted lines enclose the desired clean water region.

Figure 10a–d report on the effect of the electric field of values spanning the range *E* = 0.1–3.0 V/Å. Ion concentration (Na^+^ and Cl^−^) is at *c* = 1.39% (mass percentage). The region between the two dotted lines is the “clean water” region, from which it is desirable to obtain pure water flow. For *E* = 0.1 V/Å (Figure 10a), ions are mainly localized inside the two reject regions. Two similar peaks of correlated Na^+^ and Cl^−^ ions emerge near the “positive” bottom wall. The remaining ions throughout the channel are mainly Na^+^ ions that tend to move toward the upper wall (“negative” charged wall). By increasing the electric field to *E* = 1.0 V/Å (Figure 10b), for the same simulation duration as for the *E* = 0.1 V/Å case, *t* = 15 ns, it is noticed that the increase in the electric field magnitude has overcome the correlation between anions and cations and a new Na^+^ peak appears close to the top wall. In Figure 10c, where *E* = 2.0 V/Å, two sharp Na^+^ and Cl^−^ density peaks are observed near the bottom wall, while another Na^+^ peak is observed near the top wall, along with a small Cl^−^ density peak. The central “clean water” region is almost free from ions. Finally, in Figure 10d, the increase in the electric field value to the extreme value of *E* = 3.0 V/Å results in sharper ion peaks near the walls inside the two “reject” regions. Correlations do exist even for this *E* value, but they are weaker now.

The results shown here for ionic flow across nanochannels under the effect of various electric field values suggest that a higher *E* value accelerates the ion separation process, meaning that albeit the satisfying percentage of pure water around the channel midplane even for *E* values from 0.1 V/Å, water desalination occurs faster as the voltage increases. In real systems, there has to be a compromise between speed and energy cost.

### 3.3. Channel Length and Volumetric Flow Calculations

Channels simulated in this work are periodic along the *x*-dimension and there is no evidence of the optimal channel length to be used in a model such as the one proposed in Figure 2. To account for the optimal channel length that could yield satisfying ion separation results, the average fluid velocities, ${\bar{\upsilon }}_{f}$, have to be extracted, after simulations with duration of *t* = 20 ns. From the previous sections, it is obvious that this time is adequate to provide ion-free water flow inside the “clean water” region and could supply the proposed channel outlet with clean water in the central region.

**Figure 11:**
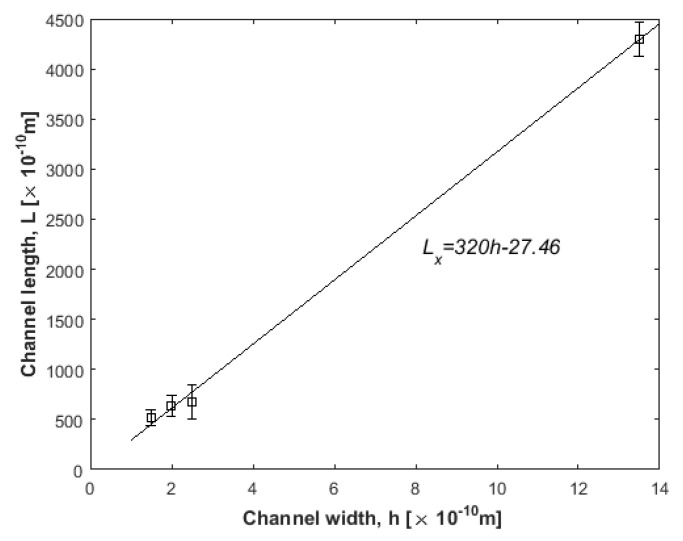
Calculated minimum channel length vs. channel width (squares) to be incorporated for the ion separation model of Figure 2. A line is a linear fit.

The minimum channel length, *L_x_*, for each channel width, *h*, investigated in this work is given by $L_{x}={\bar{\upsilon }}_{f}\cdot t$. Flow conditions include parameters *E* = 2.0 V/Å, *F_ext_* = 0.025 kcal/molÅ, and *t* = 20 ns, and results are depicted in Figure 11. It seems that there exists a linear relation between the channel width incorporated and the respective channel length, at least for the channel range studied in this work.

**Figure 12:**
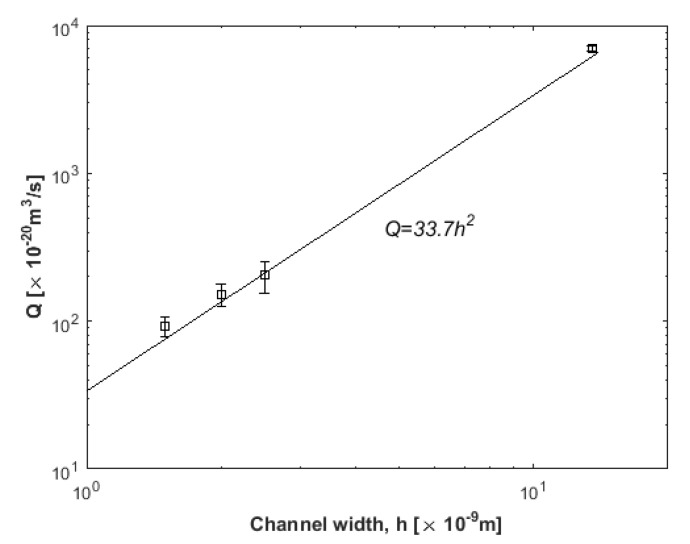
Volumetric flow rate, *Q*, vs. channel width (squares). Line is power-law least-squares fit.

From another point of view, after a comparison between the respective volumetric flow rate from the clean water region $Q=\bar{\upsilon }\times A=\bar{\upsilon }\times \left(a\times L_{y}\right)$ (*A* is the cross-sectional channel area, *a* is the clean water region width, and *L_y_* is the channel dimension on *y*-dimension) for each channel is made, results are shown in the logarithmic plot in Figure 12. Data points are organized in a power-law curve. A power-law least squares approximation of the form $Q=33.7h^{2}$ represents the data very well.

It is obvious that the flow rate of clean, desalinated water is enhanced for the larger channel under the same driving force, and this could result in better desalination performance.

## 4. Discussion

The idea of dividing the flow field and directing unwanted substances out of the solution is not new. For example, a device for sea water desalination by ion concentration polarization was fabricated at the microscale, where the flow filed is divided in two regions: one for clean water flow and the other for unwanted substances [57]. Aiming at small-scale applications, this methodology minimizes the possibility of membrane fouling and salt accumulation, as other membrane separation methods do.

Toward this direction, the present paper employs an analogous separation principle. A Poiseuille-like channel is investigated that is periodic in the *x*-direction for water/ion flows under various conditions. As shown in Figure 2, the channel outlet separates the flow region in three sub-channels; the central sub-channel is supposed to convey clean water, while the upper and the lower one carry brine that has to be rejected. Theoretical investigation has been performed, taking in mind all model parameters and flow conditions, and emphasis has been laid on whether unwanted ions have approached the wall-reject regions, leaving clean water flowing around the channel centerline, inside the region of *α* width. At the nanoscale, channel wall effects are strong, and more parameters have to be borne in mind compared to similar applications at the macroscale [34].

Numerical results are gathered in Table 3 to provide means of comparison among the different parameters exploited in this work. Starting from the small channel of *h* = 1.5 nm, for ion concentration in water *c* = 1.39% and *E* = 2.0 V/Å, maximum ion separation takes place after *t* = 20 ns. At *t* = 15 ns, only 33% and 66% separation for Na^+^ and Cl^−^ ions, respectively, is achieved. It should be clarified that at these scales, the number of ions that flow along the channel is practically small (i.e., 6 ions flowing inside 432 H_2_O molecules in the *h* = 1.5 nm channel). However, the *t* = 20 ns simulation duration time seems adequate for extracting statistically accurate results. On the other hand, by keeping ion concentration low at *c* = 1.39%, we abide by the water/salt values usually incorporated in real desalination applications. It is also of importance to note that the proposed nanochannel geometry could be part of a wider network of nanochannels where the desalination capability would increase significantly.

By increasing the channel width to *h* = 2.0 nm, for the same flow conditions, it takes less simulation time for ions (*t* = 15 ns) compared to the *h* = 1.5 nm channel to approach the two wall-reject regions. Ion rejection reaches 100% for the *h* = 2.5 nm channel at *t* = 20 ns as well. As long as the *h* = 13.5 nm channel is concerned, at *t* = 15 ns, ion rejection varies among 82–93% for all *E* values incorporated in the simulations.

Successful ion removal from the channel interior has been achieved for most cases shown here after *t* = 20 ns. The electric field value is not the sole parameter that eases ion removal; ion correlations between Na^+^ and Cl^−^ ions have a significant contribution in this process, and the results qulitatively agree with relevant findings in the literature [50].

Another basic outcome from the numerical results analysis is the fact that MD simulations, at least for the channel range used in this work, demand a minimum of *t* = 15–20 ns simulation time to extract accurate, equilibrated results. To report on such a proposed ion separation configuration at the low voltage range, the simulation time might need even more time to provide accurate results. On the other hand, for simulation purposes, one could boost the simulation speed by increasing the voltage applied across the channel. By increasing *E* values to 3.0 V/Å, it approaches the electric field magnitude that could cause electro-vaporization, i.e., it could break the chemical bonds and induce the ionization of water molecules. However, the rigid structure of water molecules is kept using the SHAKE algorithm. Similar electric field values have been also incorporated in other MD papers concerning water simulations where the SHAKE algorithm is employed [58,59]. It is emphasized that this is not suggested for real applications; it only provides qualitative simulation results.

It is also of interest to point out the difference observed in some cases between Na^+^ and Cl^−^ peaks in density profiles presented in Figure 5, Figure 7, Figure 9, and Figure 10, which means that their distribution is asymmetric throughout the channels. This behavior is attributed to wall wettability properties, as our carbon walls act as Na-ionophobic and Cl-ionophilic, as explained in detail in Section 3.1.

Conclusively, the proposed channel and outlet geometry in Figure 2 could be incorporated for novel desalination applications that could be energy efficient, without the need to construct some kind of membrane configuration, special electrodes, or a fouling-reject mechanism. Moreover, there are no Faradaic phenomena occurring, as in usual CDI applications [60]. The choice of carbon as the wall material has been made for the ease of simulations; however, it would be a low-cost choice in a real system, either as manufacturing cost or as environmental footprint [5,61]. The channel width has been found to depend linearly to the channel length that has to be incorporated. Nevertheless, this theoretical model has yet to account for many parameters, so it could be applicable in a real system in terms of investigating the optimal wall material, various ions or other unwanted substances concentration, operational temperature, and construction of the channel outlet.

Overall, having in mind the assumptions made in this paper (for example, no other chemical phenomena exist, such as H-bonding or ion hydration), it seems that there is qualitative agreement with the literature, both from simulations and an experimental point of view, and it suggests an ion separation method that could be exploited in a practical, small-scale system.

## 5. Conclusions

The ion separation model presented in this work is evaluated on salt ion rejection in Poiseuille-like flows in a way that clean water flows around the channel interior and is being gathered in the outlet. Molecular Dynamics simulations are conducted to better understand water/ion flow behavior under the combining effect of an electric field normal to the walls and an external force that drives the flow, incorporating various channel heights, at an ion concentration around 1.35–1.39%. Ion rejection is investigated for voltage values around E = 0.1–3 V/Å and reaches 100% in less than t = 20 ns for the specific channels. The flow rate of clean water is enhanced for larger channels, as expected; however, smaller channels are found to reach 100% ion removal faster. It was also found that ion correlations are important for understanding the distribution of ions across the nanochannel.

The findings reported here open a different perception for CDI-like applications, while they are in qualitative agreement with the existing literature results. In terms of exploiting the proposed theoretical ion separation system to a real-scale device, it emerges that this would be low cost, energy efficient choice, and easy to implement, without the need to anticipate for special separation membranes, special porous electrodes, or a fouling-reject mechanism as employed in CDI.

Albeit the promising results extracted by the simulations, extensive computational analysis still needs to be made, examining all possible scales from nano to macro to unlock the ion separation mechanism in the proposed configuration, estimate mass and heat transfer characteristics and power consumption, and decide on the geometrical characteristics of the proposed system that could lead to the commercialization of the method. For example, a future perspective would be the proposed system re-arrangement to a matrix of nanochannels, performing ion separation in parallel, which was integrated into a novel desalination device.

## Figures and Tables

**Table 1:** Model details. Channels of height *h* = 1.5–13.5 nm are incorporated. *L_i_* is the length of simulation box in each dimension (*i* = *x*, *y*, or *z*), a: the clean water region shown in Figure 2, N_H2O_ and N_C_: the number of H_2_O molecules and carbon atoms, respectively.

*L_x_* × *L_y_* × *L_z_*	*h*	*a*	*N*_H2O_	*N_C_*
3.0 × 3.0 × 2.5 nm	1.5 nm	0.6 nm	432	722
3.0 × 3.0 × 3.0 nm	2.0 nm	0.8 nm	594	722
3.0 × 3.0 × 3.5 nm	2.5 nm	1.0 nm	729	722
4.5 × 4.7 × 14.7 nm	13.5 nm	5.4 nm	8640	3654

**Table 2:** Potential parameters for all atomic pairs.

Atom Pair	*ε* [kcal/mol]	*σ* [Å]	Mass [a.u]
H–H	0.000	0.000	1.008
O–O	0.155	3.166	15.990
C–C	0.056	3.400	12.010
Cl–Cl	0.107	4.446	35.450
Na–Na	1.607	1.897	22.990

**Table 3:** Numerical results for every simulation case.

Simulation Parameters					Percentage of Removal	
*h* (nm)	*E* (V/Å)	*c* (%)	*t* (ns)	*F_ext_* (kcal/molÅ)	Na^+^	Cl^−^
1.5	2.0	1.39	10	0.025	0	0
1.5	2.0	1.39	15	0.025	33	66
1.5	2.0	1.39	20	0.025	100	100
2.0	2.0	1.35	10	0.025	0	0
2.0	2.0	1.35	15	0.025	100	100
2.0	2.0	1.35	20	0.025	100	100
2.5	2.0	1.37	10	0.025	60	80
2.5	2.0	1.37	15	0.025	100	80
2.5	2.0	1.37	20	0.025	100	100
13.5	0.1	1.39	15	0.025	82	90
13.5	1.0	1.39	15	0.025	93	93
13.5	2.0	1.39	15	0.025	87	87
13.5	3.0	1.39	15	0.025	93	90
